# Multi-valent pneumococcal conjugate vaccine for global health: From problem to platform to production

**DOI:** 10.1080/21645515.2022.2117949

**Published:** 2022-10-14

**Authors:** Anup Datta, Kapil Kapre, Indah Andi-Lolo, Subhash Kapre

**Affiliations:** a Bacterial Vaccine R&D, Inventprise, Inc., Redmond, WA, USA; b Process Automation & Support, Inventprise, Inc., Redmond, WA, USA; c Portfolio Management, Inventprise, Inc., Redmond, WA, USA; d Founder Chairman, Inventprise, Inc., Redmond, WA, USA

**Keywords:** Pneumococcus, pneumococcal conjugate vaccine, vaccine development, GAVI, Gates Foundation, Inventprise

## Abstract

Childhood bacterial meningitis and pneumonia represent leading causes of mortality, with the latter persisting as one of the top causes of mortality for children under 5 y of age. The prohibitive costs of developing and producing broader spectrum conjugate vaccines impact availability and affordability, resulting in a barrier to health equity and access to disease preventing vaccines, which restrict global health disease prevention efforts. Inventprise was founded in response to the need for innovation that can help reduce disease burden with improved coverage and more affordable vaccines. Inventprise 25-valent pneumococcal conjugate vaccine candidate with the patented Hz-PEG-Hz linker technology platform is expected to provide the broadest coverage against pathogenic pneumococcal serotypes encountered by populations regardless of where they live. The innovative automation technology and tightly controlled manufacturing requirements were implemented to mitigate the high capital cost for constructing a manufacturing facility in the United States, in addition to the prohibitive cost for the workforce required for running a complex plant.

## Introduction

Childhood bacterial meningitis and pneumonia represent leading causes of mortality, with the latter persisting as one of the top causes of mortality for children under 5 y of age.^1^ Two common causes of these serious diseases, *Haemophilus influenzae* serotype b (Hib) and *Streptococcus pneumoniae* (pneumococci) are responsible for tens of millions of serious illnesses and hundreds of thousands of deaths each year.^2^ While mortality is highest in low- and middle-income countries where access to adequate treatment is poor,^3^ these diseases are truly a global problem, denoted by inclusion of vaccination in 146 national immunization programs worldwide.^4^

Existing conjugate vaccines for Hib and pneumococci are staples of routine infant immunization programs worldwide. A multidisciplinary group supported by the GAVI Alliance and the Bill & Melinda Gates Foundation conducted mathematical modeling to estimate the number of expected deaths averted in 73 countries supported by the GAVI Alliance. The number of future averted deaths forecasted in this study indicated that between 2011 and 2020, Hib vaccination was expected to avert approximately 1.4 million deaths, whereas *S*. *pneumoniae* was expected to avert approximately 1.5 million deaths.^5^

While new vaccines that protect against these diseases have been introduced, there is room for improvement, both in terms of protection offered and in terms of cost-effectiveness. In low- and middle-income countries, where mortality levels are high and the burden of bacterial respiratory disease is greatest, an unmet need exists for conjugate vaccines that can offer broad protection at a reasonable price. The prohibitive costs of developing and producing broader spectrum conjugate vaccines impact availability and affordability, resulting in a barrier to health equity and access to disease preventing vaccines, which restrict global health disease prevention efforts.

## Formation of Inventprise

Inventprise was founded in response to the need for innovation that can help reduce disease burden with improved coverage and more affordable vaccines. To support this effort, Inventprise needed to develop vaccines that were more immunogenic, easier to produce and affordable at the global level. The challenges were clear, and the stakes, namely child mortality, could not be higher.

The initial efforts started in June 2013, when Inventprise set up a 600 sq. ft laboratory in Seattle. In November 2014, Inventprise moved to a larger facility of over 10,000 sq. ft space in Redmond, Washington, which included a cGMP laboratory capable of producing drug substances, although this was not adequate to produce complex vaccines. To overcome this issue, a 30,000 sq. ft lab was designed according to cGMP specifications for vaccine development, in addition to a research and development lab of 8000 sq. ft for bacterial vaccine development.

As Inventprise continued development activities of the conjugate platform, the company also began setting up collaborative partnerships with the Naval Medical Research Institute (Bethesda, MD), Kaketsuken (Japan) and the National Research Council (NRC, Canada). These collaborations brought about newer developments in vaccines and enabled a rapid development of quality analysis, which is fundamental to establishing quality requirements. Complex analytical procedures such as a multiplex ELISA system were established, which could help measure mixed antigens in a formulated vaccine dose as well as mixed antibodies generated in animals by multivalent vaccines in development studies.

Inventprise also expanded their relationship with the Bill & Melinda Gates Foundation, engaging the foundation to support their vaccine development efforts, initially through program-related grants.

## Evolution of linker technology

In 2015, the NRC in Canada requested Inventprise to develop a novel Haemophilus influenzae type A vaccine (Hia), which could potentially address the disease burden among the native population in the Arctic circle. As the required vaccine quantities were not more than 200,000 doses per annum, there were no manufacturers that wished to take on the project due to the small quantities.

The vaccine candidate for Hia needed to show a high initial immune response equivalent to outer membrane protein complex (OMPC) conjugates as well as showing a long-lasting protection, similar to vaccines conjugated with carrier proteins. Obtaining OMPC was difficult and such a conjugate would not have comparable longevity of immune responses of a regular conjugate, which meant improvements were needed to achieve the desired immune responses. Achieving immune response higher than OMPC conjugates was not possible with regular conjugation methods like 1-cyano-4-dimethylaminopyridinium tetrafluoroborate (CDAP) or reductive amination. Considering this issue, Inventprise scientists determined that a longer link between the polysaccharide and carrier protein could help. An adipic acid dihydrazide (ADH) linker was evaluated and showed improvement, but the results were not comparable to OMPC conjugates. A review of published literature concluded in the selection of a Hydrazine-Polyethylene Glycol-Hydrazine linker (Hz-PEG-Hz linker) which indicated that the PEG addition could help in obtaining a high immune response. The published preclinical studies^6^ showed that polysaccharide-conjugate vaccines that include PEG elicit significantly higher IgG titers and antibody avidity compared to the same polysaccharide-conjugate without PEG in the conjugate. The immune enhancing effect of the PEG linker is thought to be due to an increase in half-life of the polysaccharide conjugate. It could also reduce steric hindrance by the protein carrier due to the linker length and polysaccharide sizing. An additional rationale behind selecting the homo-bifunctional Hz-PEG-Hz linker is the PEG’s positive safety profile in approved PEG biopharmaceuticals. Hydrazine has previously been used as linker for conjugation in the meningococcal conjugate vaccine, MenAfriVac, of which more than 500 million doses have been administered without any safety concerns.^7^ The PEG component has also been used as a safe component conjugated to antibodies and other biologicals.

Following the selection of the Hz-PEG-Hz linker, Inventprise team conducted a series of proof-of-concept animal studies by evaluating a novel bivalent conjugate with two polysaccharides, Hib and Hia, linked to a common carrier protein (CRM_197_). The idea was to compare Hib sera results in both conjugates with Hib OMPC. If both Hib components were higher, then one could conclude that accompanying Hia conjugate would also be showing an equivalent response similar to an OMPC conjugate. The formulated products with Alum adjuvant were tested against an existing standard-of-care Hib OMPC conjugate and conducted in New Zealand white female rabbits. Three groups of rabbits each received a Hia-Hz-PEG-Hz-conjugate, Hia-Hz-PEG-Hz-CRM_197,_ Hz-PEG-Hz-Hib conjugate or Hib-OMPC conjugate. The serum samples were tested using a multiplex bead-based ELISA to quantify IgG concentrations in the rabbit serum samples. Surprisingly, the Hia-Hib-PEG-Hz Hib component gave a higher immune response to both formulations than the Hib OMPC product. This indicated that the Hia-Hz-PEG-Hz conjugate would match an OMPC conjugate should one be made as an individual conjugate. This approach successfully met both criteria and appeared to have highly immunogenic antibodies against Hia.

Having established the patented Hz-PEG-Hz linker platform with Hia vaccine, Inventprise conducted pre-clinical evaluation in New Zealand white female rabbits comparing the Inventprise 6-valent PCV against Prevnar 13®. The rabbits were primed on day 0 and boosted on day 14, with sera collected on day 14 and day 28.

The serum samples were tested using a multiplex bead-based ELISA to quantify IgG concentrations in the rabbit serum samples. IgG antibody concentrations for test vaccine formulations were calculated relative to the 007sp reference standard obtained from the Center for Biologics Evaluation and Research, US Food and Drug Administration. Inventprise has created an in-house QC rabbit serum (25-V) based on the Reference standard 007sp, which is used in every assay to verify the assay variability. Standard value assigned arbitrarily 10 ug/ml on serotype 6C, 15A, 16F, 24F and 35B in the assays due to lack of values in the International Standard serum 007sp. This formulation gave a higher immune response when compared to Prevnar 13®.

**Figure 1.**
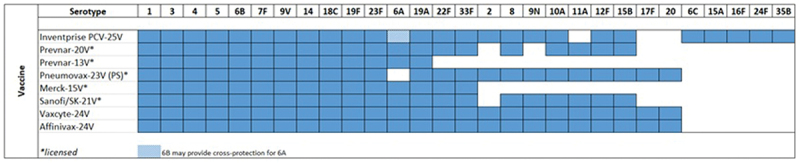
Serotype distributions across various pneumococcal vaccines.

Following the promising result from Inventprise 6-valent PCV, the team embarked on a series of preclinical studies evaluating Inventprise formulated PCVs with a greater number of serotype conjugates to determine how much one could increase the number of serotypes to create a broad-based vaccine. Encouraged by the responsiveness of the novel platform technology, the team proceeded with a preclinical evaluation of a 25-valent formulation developed by Inventprise against Prevnar 13®. Serotypes were selected based on their global disease burden (Figure 1). The comparative IgG analysis from the various preclinical studies of Inventprise’s higher valency PCVs with the Hz-PEG-Hz linker elicited a high serotype-specific immune response compared to both Prevnar 13® and to Inventprise no-linker formulation. Publication is under preparation outlining the results from these pre-clinical studies.

With other high valent vaccines, the experience to date has shown that, when the number of conjugates increased in a formulation, there was a drop in potency of the polysaccharide conjugates and an increase in antibodies to CRM_197_ limiting the increase in serotype numbers.^8^ However, this was not observed in the Inventprise linker-based formulation despite the increase in serotype numbers, and there was no increase in the carrier protein antibodies. The linker technology not only helped maintain the high titers of all conjugates in a formulation irrespective of the increase in the serotypes but also limited carrier protein antibodies increase. This technology helped create a broad-based pneumococcal conjugate, which led to the development of the PCV 25 vaccine candidate.

**Figure 2.**
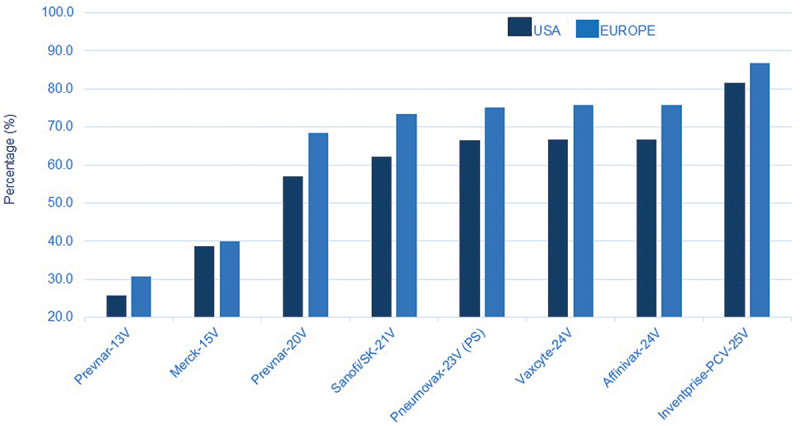
Estimate coverage based on circulating serotypes during the 2017 epidemiological years (in US^9^ and Europe^10^) in all age groups. This also would follow in the rest of the world as these stereotypes are present in other parts of the world.

As outlined in Figure 2 the serotypes in the Inventprise 25-valent pneumococcal conjugate covers ≥80% of the circulating serotypes in the USA and Europe, based on the survey from the 2017 epidemiological years. With the Hz-PEG-Hz linker technology platform, the vaccine candidate is expected to provide the broadest coverage against pathogenic pneumococcal serotypes encountered by populations regardless of where they live. The platform is scalable to a >32-valent PCV to further expand serotype coverage if necessary. Based on the promising results seen in the previous preclinical studies, Inventprise moved into an IND-enabling GLP toxicology study with the 25-valent formulation.

## Production to solve a global problem

Having formulated a 25 valent conjugate vaccine, there was a necessity to establish methods to test the drug substance (the 25 valent pneumococcal conjugate formulation) and the drug product. The Inventprise team proceeded to refine all the necessary analytical methods needed for this complex product, which enabled the establishment of the production methods to make this as an affordable vaccine. This effort led toward the designing and establishment of a suitable plant for large-scale manufacturing. Inventprise converted a large existing warehouse into an 80,000 sq ft GMP manufacturing facility for drug substances and drug product production. The design and implementation were entirely done in-house, with the goal of having the large-scale batches available for phase 3 study.

The capital cost for constructing a manufacturing facility in the United States is high, in addition to the prohibitive cost for the workforce required for running a complex plant. The product, however, needed to be manufactured at an affordable price. This was a formidable challenge. Another challenge was to implement process automation and process step reduction.

After refinement of each of the working steps, such as ultrafiltration, bacterial mass removal, membrane filtration, column chromatography steps, etc., automation steps were designed to reduce headcount to operate the manufacturing facility. The goal was to ensure that the process, from fermentation to obtaining the purified product, could be conducted in a seamless way, to help produce a greater number of lots per unit of time, increasing output and by reducing the plant size to reduce capital cost and the dependency on the workforce. The design, refinement and implementation were all performed in-house at Inventprise, and by bringing this together created the possibility of making a complex vaccine development more affordable.

**Figure 3.**
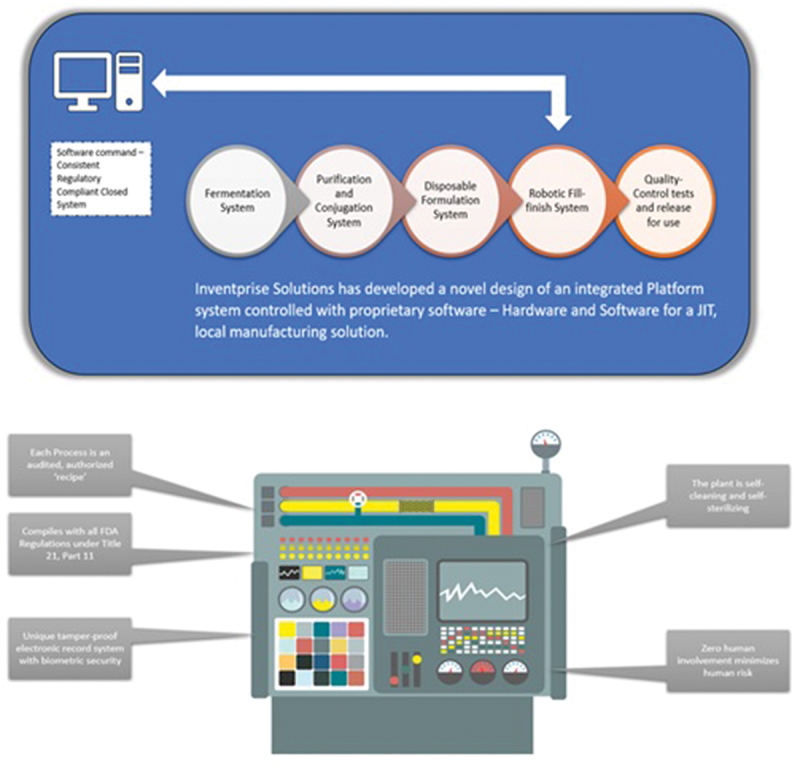
Inventprise solutions has developed a novel design of an integrated platform system controlled with proprietary software – hardware and software for a JIT, local manufacturing solution.

The design depicted in Figure 3 was incorporated into the facility, adhering to current GMP standards, with one wing for the polysaccharide manufacturing area and another for the manufacture of the carrier protein. A third workspace had conjugation plant and area for formulation. The design of the production space was carefully considered, incorporating an efficient process engineering flow for the multi-step process of fermentation, purification and conjugation. The organizational structure of the rooms promotes clean spaces and minimizes interaction and contamination risks between reagents at various stages within the manufacturing process. The innovative automation technology and tightly controlled manufacturing requirements like raw materials, consumables, etc., would help implement a high-throughput manufacturing facility adhering to GMP standards, enabling high-speed large-scale manufacturing.

Inventprise hopes to produce this vaccine at an affordable price for the global health market, with the underlying goal of supplying large quantity of this vaccine to reduce child mortality. This platform can also be used for making other vaccines that are needed by countries that benefit from UNICEF and GAVI vaccine procurement and funding programs.

## Conclusion

From a small beginning as a small research idea, to the discovery of a stable linker platform with the potential to provide expanded valency protection without reducing serotype-specific immunogenicity, Inventprise has navigated the complex and daunting challenges of starting a life science company. These efforts described could help researchers to establish their products following a similar path. The narrative is more to encourage aspiring scientific researchers to enter the realm of manufacturing for their developed products. The key elements include having a novel idea, establishing dependable assays and analytical procedures, designing a low-cost plant and machinery through automation as the main driver to establish a workable manufacturing plant from a research idea.
